# Supplementary material for: A novel hole performance index to evaluate the hole geometry and drilling time in the electrochemical drilling process
Source: PLoS One. 2024 Dec 3;19(12):e0314236. doi: 10.1371/journal.pone.0314236 (PMC11614253; doi:10.1371/journal.pone.0314236)

PLOS ONE

A NOVEL HOLE PERFORMANCE INDEX TO EVALUATE THE HOLE GEOMETRY AND DRILLING TIMEIN THE ELECTROCHEMICAL DRILLING PROCESS

--Manuscript Draft--

**Manuscript Number:**	PONE-D-24-24552
**Article Type:**	Research Article
**Full Title:**	A NOVEL HOLE PERFORMANCE INDEX TO EVALUATE THE HOLE GEOMETRY AND DRILLING TIMEIN THE ELECTROCHEMICAL DRILLING PROCESS
**Short Title:**	A Novel Hole Performance Index and Drilling Time Evaluation of Electrochemical Drilled Holes
**Corresponding Author:**	Hacı Bekir Özerkan<br>Gazi Üniversitesi: Gazi Universitesi<br>ÇANKAYA, ANKARA TURKEY
**Keywords:**	Electrochemical Drilling, Hole Performance Index, Hole Geometry, Drilling Time, ANOVA, Optimization
**Abstract:**	Electrochemical Drilling (ECD) is a non-traditional manufacturing process designed to produce holes of varying cross-sections that do not require additional finishing operations on workpieces that are difficult to drill due to their hardness and internal structure. This paper examines the impact of process variables, such as machining voltage, electrolyte concentration, electrode rotational speed, electrolyte flushing pressure and workpiece material, on the novel hole performance index HPI in the electrochemical drilling process. It was found that the HPI was a suitable metric for evaluating the hole geometry and drilling time for varying machining parameters and workpiece materials. The significance of each machining parameter and workpiece material on the HPI was ascertained using the analysis of variance (ANOVA) method. The study used signal-to-noise S/N ratio analysis to determine the optimal machining parameters. The results indicated that the workpiece material and machining voltage were highly effective parameters for HPI. The confirmation tests indicated that it is possible to minimize HPI significantly by using the proposed statistical method.
**Order of Authors:**	Nihat Tosun
	Hacı Bekir Özerkan
	Can Çoğun
	Raja Venkatesan, Prof. Dr.
**Additional Information:**	
**Question**	**Response**
**Financial Disclosure**<br><br>Enter a financial disclosure statement that describes the sources of funding for the work included in this submission. Review the submission guidelines for detailed requirements. View published research articles from *PLOS ONE* for specific examples.<br><br>This statement is required for submission and **will appear in the published article** if the submission is accepted. Please make sure it is accurate.	Yes

Powered by Editorial Manager® and ProduXion Manager® from Aries Systems Corporation

**Funded studies**<br>Enter a statement with the following details:<br>• Initials of the authors who received each award<br>• Grant numbers awarded to each author<br>• The full name of each funder<br>• URL of each funder website<br>• Did the sponsors or funders play any role in the study design, data collection and analysis, decision to publish, or preparation of the manuscript?<br><br>Did you receive funding for this work?	
Please add funding details.<br>as follow-up to "**Financial Disclosure**<br><br>Enter a financial disclosure statement that describes the sources of funding for the work included in this submission. Review the submission guidelines for detailed requirements. View published research articles from *PLOS ONE* for specific examples.<br><br>This statement is required for submission and **will appear in the published article** if the submission is accepted. Please make sure it is accurate.<br><br>**Funded studies**<br>Enter a statement with the following details:<br>• Initials of the authors who received each award<br>• Grant numbers awarded to each author<br>• The full name of each funder<br>• URL of each funder website<br>• Did the sponsors or funders play any role in the study design, data collection and analysis, decision to publish, or preparation of the manuscript?<br><br>Did you receive funding for this work?"	The author, R.V., would like to thank the National Research Foundation of Korea (NRF), which was funded by the Ministry of Education
Please select the country of your main research funder (please select carefully as in some cases this is used in fee	KOREA, REPUBLIC OF - KR

calculation).<br>as follow-up to "**Financial Disclosure**<br><br>Enter a financial disclosure statement that describes the sources of funding for the work included in this submission. Review the submission guidelines for detailed requirements. View published research articles from *PLOS ONE* for specific examples.<br><br>This statement is required for submission and **will appear in the published article** if the submission is accepted. Please make sure it is accurate.<br><br>**Funded studies**<br>Enter a statement with the following details:<br>• Initials of the authors who received each award<br>• Grant numbers awarded to each author<br>• The full name of each funder<br>• URL of each funder website<br>• Did the sponsors or funders play any role in the study design, data collection and analysis, decision to publish, or preparation of the manuscript?<br><br>Did you receive funding for this work?"	
**Competing Interests**<br><br>Use the instructions below to enter a competing interest statement for this submission. On behalf of all authors, disclose any competing interests that could be perceived to bias this work—acknowledging all financial support and any other relevant financial or non-financial competing interests.<br><br>This statement is required for submission and **will appear in the published article** if the submission is accepted. Please make sure it is accurate and that any funding sources listed in your Funding Information later in the submission form are also declared in your Financial Disclosure	The authors have declared that no competing interests exist.

statement.<br><br>View published research articles from *PLOS ONE* for specific examples.<br><br>**NO authors have competing interests**<br><br>Enter: *The authors have declared that no competing interests exist.*<br><br>**Authors with competing interests**<br><br>Enter competing interest details beginning with this statement:<br><br>*I have read the journal's policy and the authors of this manuscript have the following competing interests: [insert competing interests here]*<br><br>* typeset	
**Ethics Statement**<br><br>Enter an ethics statement for this submission. This statement is required if the study involved:<br><br>• Human participants<br>• Human specimens or tissue<br>• Vertebrate animals or cephalopods<br>• Vertebrate embryos or tissues<br>• Field research<br><br>Write "N/A" if the submission does not require an ethics statement.<br><br>General guidance is provided below. Consult the submission guidelines for detailed instructions. **Make sure that all information entered here is included in the Methods section of the manuscript.**	N/A

Powered by Editorial Manager® and ProduXion Manager® from Aries Systems Corporation

**Format for specific study types**<br><br>**Human Subject Research (involving human participants and/or tissue)**<br>• Give the name of the institutional review board or ethics committee that approved the study<br>• Include the approval number and/or a statement indicating approval of this research<br>• Indicate the form of consent obtained (written/oral) or the reason that consent was not obtained (e.g. the data were analyzed anonymously)<br><br>**Animal Research (involving vertebrate animals, embryos or tissues)**<br>• Provide the name of the Institutional Animal Care and Use Committee (IACUC) or other relevant ethics board that reviewed the study protocol, and indicate whether they approved this research or granted a formal waiver of ethical approval<br>• Include an approval number if one was obtained<br>• If the study involved *non-human primates*, add *additional details* about animal welfare and steps taken to ameliorate suffering<br>• If anesthesia, euthanasia, or any kind of animal sacrifice is part of the study, include briefly which substances and/or methods were applied<br><br>**Field Research**<br><br>Include the following details if this study involves the collection of plant, animal, or other materials from a natural setting:<br>• Field permit number<br>• Name of the institution or relevant body that granted permission	
**Data Availability**<br><br>Authors are required to make all data underlying the findings described fully available, without restriction, and from the time of publication. PLOS allows rare exceptions to address legal and ethical concerns. See the PLOS Data Policy and FAQ for detailed information.	Yes - all data are fully available without restriction

Powered by Editorial Manager® and ProduXion Manager® from Aries Systems Corporation

A Data Availability Statement describing where the data can be found is required at submission. Your answers to this question constitute the Data Availability Statement and **will be published in the article**, if accepted.<br><br>**Important:** Stating 'data available on request from the author' is not sufficient. If your data are only available upon request, select 'No' for the first question and explain your exceptional situation in the text box.<br><br>Do the authors confirm that all data underlying the findings described in their manuscript are fully available without restriction?	
**Describe where the data may be found in full sentences. If you are copying our sample text, replace any instances of XXX with the appropriate details.**<br><br>• If the data are **held or will be held in a public repository**, include URLs, accession numbers or DOIs. If this information will only be available after acceptance, indicate this by ticking the box below. For example: *All XXX files are available from the XXX database (accession number(s) XXX, XXX.*).<br>• If the data are all contained **within the manuscript and/or Supporting Information files**, enter the following: *All relevant data are within the manuscript and its Supporting Information files.*<br>• If neither of these applies but you are able to provide **details of access elsewhere**, with or without limitations, please do so. For example:<br><br>*Data cannot be shared publicly because of [XXX]. Data are available from the XXX Institutional Data Access / Ethics Committee (contact via XXX) for researchers who meet the criteria for access to confidential data.*<br><br>*The data underlying the results presented in the study are available from (include the name of the third party*	All relevant data are within the manuscript and its Supporting Information files.

and contact information or URL).<br>• This text is appropriate if the data are owned by a third party and authors do not have permission to share the data.<br><br>* typeset	
Additional data availability information:	Tick here if the URLs/accession numbers/DOIs will be available only after acceptance of the manuscript for publication so that we can ensure their inclusion before publication.

Manuscript
Click here to access/download;Manuscript;renamed_2f4a0.docx

A NOVEL HOLE PERFORMANCE INDEX TO EVALUATE THE HOLE GEOMETRY AND DRILLING TIME IN THE ELECTROCHEMICAL DRILLING PROCESS

[1]Tosun, N, [2]Özerkan H.B, [3]Çoğun, C, [4]Raja Venkatesan

(1) Mechanical Engineering Department, Fırat University, Elazığ, Turkey, Orcid:0000-0002-6211-5044

(2) Mechanical Engineering Department, Gazi University, Ankara, Turkey, Orcid: 0000-0002-7214-9985

(3) Mechatronics Engineering Department, Çankaya University, Ankara, Turkey, Orcid: 0000-0001-5045-2767

(4) School of Chemical Engineering, Yeungnam University, Gyeongsan 38541, Republic of Korea; e-mail: rajavenki@yu.ac.kr

Abstract- Electrochemical Drilling (ECD) is a non-traditional manufacturing process designed to produce holes of varying cross-sections that do not require additional finishing operations on workpieces that are difficult to drill due to their hardness and internal structure. This paper examines the impact of process variables, such as machining voltage, electrolyte concentration, electrode rotational speed, electrolyte flushing pressure and workpiece material, on the novel hole performance index *HPI* in the electrochemical drilling process. It was found that the *HPI* was a suitable metric for evaluating the hole geometry and drilling time for varying machining parameters and workpiece materials. The significance of each machining parameter and workpiece material on the *HPI* was ascertained using the analysis of variance (ANOVA) method. The study used signal-to-noise S/N ratio analysis to determine the optimal machining parameters. The results indicated that the workpiece material and machining voltage were highly effective parameters for *HPI*. The confirmation tests indicated that it is possible to minimize HPI significantly by using the proposed statistical method.

Article Highlights

The Hole Performance Index (HPI) is a suitable metric for evaluating hole geometry and drilling time.

Analyzed using analysis of variance (ANOVA) method to determine the significance of each parameter.

Workpiece material and machining voltage are highly effective for the Hole Performance Index (HPI).
Keywords: Electrochemical Drilling, Hole Performance Index, Hole Geometry, Drilling Time, ANOVA, Optimization.

Nomenclature

d_e	Electrode diameter (mm)
h	Hole depth (mm)
n_e	Electrode rotational speed (rpm)
t_m	Hole drilling time (min)
C_e	Electrolyte salt concentration (g/L)
A_e	Electrode cross-sectional area (mm^2)
A_{hole}	Hole cross-sectional area (mm^2)
L_r	The length of the right-side wall profile (mm)
L_l	The length of the left-side wall profile (mm)
P_f	Electrolyte flushing pressure (bar)
V_m	Machining voltage (V)
ECD	Electrochemical drilling
HACI	The hole shape conformity index
HPI	Hole performance index
HWPCI	Hole wall profile conformity index
PM	Powder metallurgy

1. Introduction

Electrochemical hole drilling (ECD) is a nonconventional manufacturing approach developed to create holes with various cross-sections. The ECD method involves separating the cathode tool electrode (shortly, "electrode") from the anode workpiece by a small gap and rapidly dissolving the anode (anodization) by applying an electrical current to the electrolyte. An electrolyte is flushed from the centre of the insulated metal tubular tool

towards the workpiece to remove debris from the processing medium and cool the medium during operation. In deep drillings, ionic and solid residues from electrolysis can obstruct the processing medium, slowing the drilling process and creating shapeless holes with non-uniform and wavy walls. As a result, achieving dimensional and geometrical hole accuracy remains the primary objective of this method.

Table 1 summarizes the hole performance outputs used in research studies [1-30] to enhance the ECD process performance. The average diameter and radial overcut were the research's most commonly used performance outputs. Other frequently used outputs include inlet/exit diameters, taper (conicity), material removal rate, roundness, wall surface roughness, depth, and drilling stability of the hole. The rarely used outputs were drilling time, machining gap, current, electrode wear, wall area, wall profile length, bottom geometry (for blind holes), and wall delamination (for composite workpieces). The performance outputs related to hole dimensions (average diameter, average radial overcut, taper and hole entry and exit diameter) and hole geometry were examined using tools such as optical microscopes equipped with a camera, laser scanning microscopes, digital microscopes, 3D-coordinate measuring machines (CMM), and image processing software. The hole dimensions were generally measured at specific points (i.e., predetermined depths) on the cross-sectional hole images. The profilometers and SEM were used to analyze the holes' surface characteristics, such as roughness and texture. These outputs were sometimes combined to create new indexes defining hole quality and drilling performance in ECD. The study by Bilgi et al. [16, 17] introduced two indexes to define hole quality. One is the radial overcut, and the other is the ratio of the material removal rate to the electrode feed speed. The Ayhan et al. study [30] used indexes to evaluate the geometrical characteristics of the holes, including depth, cross-sectional area and profile length. Another index was first introduced by one of the authors of this study [31] to represent the geometric quality of the machined cavity in die-sinking electric discharge machining.

The literature survey (Table 1) shows that the workpiece materials studied in the research could be more challenging to machine conventionally. These materials include Ni, Ni alloys, stainless steels (typically SS 304), Inconel, Fe-Ni alloy, Ti alloys (typically Ti6Al4V), Cr-Ni alloys, HSS, ZrO_2 ceramic, WC-Co alloys, Al-Ti alloy, hard steel alloys, valve steels and Al-TiB_2 composite.

The literature survey findings on the ECD process are as follows:

a) The drilling time is a critical performance output that determines the cost and production rate of holes in ECD. Studies have been conducted to determine hole dimensional and geometrical quality using the average diameter, radial overcut, the difference between the inlet and exit diameters, taper (conicity), roundness, and wall surface roughness measurements of the hole. However, studies have yet to be conducted on defining novel performance indexes that combine hole geometry with drilling time and statistical techniques to understand the impact of drilling time and process parameters on these new performance indexes.

b) The non-uniform and wavy hole walls, caused by electrochemical machining instabilities, have not been considered in assessing the geometric quality of the holes.

c) The drilled workpiece materials in the research are hard-to-machine (by conventional chip removal processes) materials. However, there is still considerable scope for using materials such as hard-to-machine parts due to strain hardening under cutting forces, as exemplified by Hadfield Steel. Furthermore, conductive powder metallurgy (PM) parts exhibit distinctive anodization characteristics due to their unique compacted and sintered powder structure.

Considering the gaps in the literature, this study aims to investigate the performance of a novel Hole Performance Index *HPI* in evaluating the hole geometry and hole drilling time in ECD with machining parameters for different workpiece materials and optimizing machining settings for minimum *HPI*. The machining parameters include machining voltage, electrode rotational speed, electrode flushing pressure, and electrolyte concentration. The workpiece materials studied are AISI 1040 steel, PM part, and Hadfield steel. The study also evaluated the impact of machining parameters on *HPI* using Analysis of Variance (ANOVA) to interpret the obtained experimental findings statistically. The signal-to-noise ratio analysis was utilized to obtain the parameters for the minimum *HPI*.

Table 1 Hole performance outputs used in ECD research studies

Reference	Workpiece	Hole Performance Measures																
		Average diameter	Radial overcut	Inlet, exit diameter	Taper	Roundness	Area	Profile length	Wall surface roughness	Wall delamination	Material removal rate	Depth	Machining current	Drilling time	Drilling stability	Electrode wear	Gap distance	Blind hole bottom geometry
Ahn et al. [1]	SS 304	√																
Fan and Hourng [2]	Ni alloy	√																
Zhu et al. [3]	Stainless steel alloy	√											√		√			
Wang et al. [4]	Hard steel alloy												√		√			
Wang et al. [5]	0Cr18Ni9		√	√											√			
Fan and Hourng [6]	SS 304		√		√													
Fan et al. [7]	Ni		√		√													
Mithu et al. [8]	Ni			√							√			√	√			
Choi et al. [9]	WC-Co alloy				√				√					√		√	√	
Fang et al. [10]	SS 304			√								√						
Wang et al. [11]	SS 304			√														
Zeng et al. [12]	Ti alloy											√					√	

Chandrasekhar and Prasad [13]	Al-TiB Composite		√							√	√							
Hung et al. [14]	Stainless steel		√	√														
Sharma et al. [15]	Inconel																	
Bilgi et al. [16]	Super alloy		√								√							
Bilgi et al. [17]	Ni super alloy		√								√							
Jain et al. [18]	Fe-Ni (incoloy 800)		√		√						√			√				
Zhang et al. [19]	Ti alloy, Ni alloy															√	√	√
Ali et al. [20]	Inconel	√			√													
Chavoshi [21]	VV50 (valve steel)		√						√		√							
Tajdari and Chavoshi	SS 304		√															
Lilong et al. [23]	SS 304	√		√														
Manikandan et al. [24]	SS 304										√							
Deka and Fangueir [25].	Steel	√										√						
Yang et al. [26]	ZrO2 ceramic	√				√												

Safaei et al. [27]	Ti6Al4V	√				√			√									
Kumar et al. [28]	Ti6Al4V	√				√			√									
Özerkan and Çoğun [29]	PM steel	√	√	√	√													
Ayhan et al. [30]	HSS						√	√				√						

2. Experiments

The experiments were conducted using the setup developed in a previous study (Figure 1) by the authors [32]. 0.5 mm diameter brass hollow electrodes were used to drill through holes in workpiece materials. The outer cylindrical surface of the electrode is coated with cyanoacrylate, except for the 1 mm distance from the tip, to prevent taper formation caused by excessive machining of the hole wall as the electrode penetrates.

A NaCl-water solution was used as an electrolyte due to its high electrochemical reactivity, affordability and availability. The electrolyte was flushed through the electrode hole.

The experiments tested four machining parameters: machining voltage V_m, electrolyte concentration C_e, electrode rotational speed n_e and electrolyte flushing pressure P_f. The machining settings used are shown in Table 2.

The workpiece materials were AISI 1040 steel, Hadfield steel and sinter-hardened FLN2-4405 PM steel. The composition of AISI 1040 steel was 0.75% Mn, 0.38% C, 0.20% Si, 0.07% Cr, 0.01% Mo, 0.1% Ni, 0.15% Cu by weight, balance Fe. The composition of Hadfield steel was 13.6% Mn, 1.08% C, 0.72% Cr, 0.62% Si, 0.3% Ni, 0.26% Mo, and 0.18% Cu by weight, balance Fe. The sinter-hardened FLN2-4405 PM steel had a composition of 1% Ni, 0.65% Mo, 0.4% C and 0.05% Mn by weight, balance Fe. A through hole was drilled at the junction of AISI 1040 steel and Hadfield steel workpieces, measuring 50 mm x10 mm x 8 mm, and PM steel workpieces, measuring 50 mm x 7 mm x14 mm, which allowed for the study of the hole geometries without cutting the workpieces in half.

A full factorial design of experiments was used to evaluate the effect of workpiece materials and machining parameters on *HPI* (Table 3). The drilling experiment was performed three times to ensure the reliability of the results, and the measurements were averaged.

Table 2 Machining settings

Machining parameter	Level		
	1	2	3
Workpiece material *M*	AISI 1040	Hadfield	PM
Machining voltage V_m (V)	4.5	5.5	6.5
Electrode rotational speed n_e (rev/min)	200	400	-
Electrolyte flushing pressure P_f (bar)	20	40	-
Electrolyte concentration C_e (g/L)	100	125	150

Table 3 Experimental design and results

Machining voltage V_m (V)	Electrode rotational speed n_e (rpm)	Electrolyte flushing pressure P_f (bar)	Electrolyte concentration C_e (g/L)	*HGI*			*HPI*			Remarks
				AISI 1040 steel	Hadfield steel	PM steel	AISI 1040 steel	Hadfield steel	PM steel	
4.5	200	20	100	1.74	2.31	2.66	7.85	21.68	11.61	
4.5	400	20	100	1.82	2.83	2.37	7.73	23.34	9.83	Figure 2i
4.5	200	40	100	1.87	2.79	2.39	7.72	20.59	9.20	Figure 2a
4.5	400	40	100	1.95	2.82	2.06	7.55	20.10	7.51	Figure 2f
4.5	200	20	125	2.11	3.38	2.17	8.71	22.82	6.81	Figure 2b
4.5	400	20	125	1.84	2.58	2.04	6.88	14.85	5.98	
4.5	200	40	125	2.03	2.86	1.99	7.12	17.50	5.81	
4.5	400	40	125	2.12	2.80	2.26	7.14	16.81	5.80	
4.5	200	20	150	2.07	3.74	2.60	7.25	21.03	6.68	Figure 2c
4.5	400	20	150	1.92	3.63	2.46	6.24	20.41	5.45	Figure 2d (Hadfield), Figure 2g (AISI1040)
4.5	200	40	150	2.12	2.86	2.53	7.16	16.11	5.97	
4.5	400	40	150	1.95	4.10	2.77	6.09	21.51	5.94	
5.5	200	20	100	1.87	2.87	2.18	6.30	13.62	5.14	
5.5	400	20	100	1.99	3.41	2.39	5.96	20.47	5.29	
5.5	200	40	100	2.20	2.53	2.42	7.16	10.75	5.18	
5.5	400	40	100	2.34	2.60	2.04	7.60	10.89	4.07	Figure 2j
5.5	200	20	125	2.06	2.62	1.80	5.92	10.81	4.11	
5.5	400	20	125	1.72	2.15	2.50	4.94	8.61	4.82	Figure 2e
5.5	200	40	125	2.25	2.41	2.71	6.46	9.64	5.04	
5.5	400	40	125	2.38	2.70	2.16	6.24	9.94	3.55	
5.5	200	20	150	2.24	2.20	2.14	6.16	8.26	3.82	
5.5	400	20	150	1.88	3.01	1.97	4.95	9.02	2.95	

5.5	200	40	150	2.12	2.59	2.50	5.29	9.40	3.75	
5.5	400	40	150	2.32	2.43	2.26	5.22	8.81	3.23	
6.5	200	20	100	1.87	2.24	2.30	5.60	7.56	4.60	
6.5	400	20	100	2.35	2.27	1.80	6.76	7.37	3.48	
6.5	200	40	100	1.92	2.69	2.49	5.52	8.42	4.79	
6.5	400	40	100	1.82	2.62	2.62	4.78	8.17	4.87	
6.5	200	20	125	2.26	2.74	2.59	5.64	8.56	4.99	Figure 2h
6.5	400	20	125	2.22	3.78	2.48	5.82	11.81	3.72	
6.5	200	40	125	2.05	2.90	2.30	5.13	8.51	3.94	Figure 2k
6.5	400	40	125	2.32	2.94	2.18	5.81	8.81	3.12	
6.5	200	20	150	1.95	3.07	2.65	4.69	9.22	4.54	
6.5	400	20	150	1.80	2.70	2.64	4.51	8.10	3.77	
6.5	200	40	150	1.96	3.14	2.28	4.40	9.42	3.91	
6.5	400	40	150	1.95	2.47	2.73	4.22	7.40	3.90	

Fig. 1 Electrochemical drilling setup [32]

3. Results

3.1. Experimental Hole Geometries

The hole cross-sectional geometries (Figure 2) and drilling times were experimentally obtained for the machining settings and workpiece materials. The findings are as follows:

1) The hole diameter was maximum at the entry section and minimum at the exit section of the workpiece material.

2) Localized diameter variations and non-uniform (typically wavy) wall geometries were observed in some hole sections. Both formations were attributed to the agglomeration of the residues from the dissolution reaction and the clogging of the machining zone during the process. This resulted in a short term increase in the current value and the workpiece removal rate [33]. Another factor that was effective in causing diameter changes was the deterioration of the electrode insulation.

3) In all experiments, the hole diameters were larger than the electrode diameter.

4) Drilling time decreased as the V_m, C_e, P_f, and n_e settings increased for all the three workpiece materials tested.

5) Hole diameters increased for Hadfield and AISI 1040 steels but decreased for PM steel as n_e increased.

6) The hole drilling time of the PM steel was less than that of the AISI 1040 and Hadfield steel samples due to less clogging of the machining zone and fewer interruptions during machining. The Hadfield steel had the longest drilling times.

Fig. 2 Sample Hole Geometries for various machining settings and workpiece materials. **a)** Hadfield/4.5 V/200 rpm/40 bar/100 g/L, h=8 mm, **b)** Hadfield/4.5 V/200 rpm/20 bar/125 g/L, h=8 mm, **c)** Hadfield/4.5 V/200 rpm/20 bar/150 g/L, h=8 mm, **d)** Hadfield/4.5 V/400 rpm/20 bar/150 g/L, h=8 mm, **e)** Hadfield/5.5 V/400 rpm/20 bar/125 g/L, h=8 mm, **f)** AISI1040/4.5 V/400 rpm/40 bar/100 g/L, h=8 mm, **g)** AISI1040/4.5 V/400 rpm/20 bar/150 g/L, h=8 mm**, h)** AISI1040/6.5 V/200 rpm/20 bar/125 g/L, h=8 mm, **i)** PM/4.5 V/400 rpm/20 bar/100 g/L, h=14 mm, **j)** PM/5.5 V/400 rpm/40 bar/100 g/L, h=14 mm, k) PM/6.5 V/200 rpm/40 bar/125 g/L, h=14 mm

3.2. Definition of Hole Geometry and Drilling Performance Evaluation Indexes

One of the distinctive aspects of this study is the development of novel indexes, listed below, for evaluating hole geometry and drilling performance.

The Hole Shape Conformity Index *HACI* measures the conformity between the hole cross-sectional area A_{hole} and the electrode cross-sectional area A_e. A_e is the product of the electrode diameter d_e and the hole depth h (Figure 3). The *HACI* formula is

$$\mathrm{HACL} = \mathrm{A_{hole}}/\mathrm{A_e} \tag{1}$$

In the ECD process, the *HACI* is always greater than one because the A_{hole} is always larger than the A_e due to the electrochemical dissolution of the sidewall of the hole. The *HACI* approaches 1 when the A_{hole} of the hole is close to d_e.

The Hole Wall Profile Conformity Index *HWPCI* measures the degree of waviness of the hole walls, indicating the deviation of the hole wall geometry from a perfect cylinder geometry. The length of the right and left-side wall profiles of the drilled hole cross-section are represented by L_r and L_l, respectively (Figure 3). The formula for *HWPCI* is

$$\mathrm{HWPCL} = (\mathrm{L_l}+\mathrm{L_r})/2\mathrm{h} \tag{2}$$

The *HWPCI* is always greater than one because the L_l or L_r value is always greater than the h due to wall irregularities and wavinesses. A *HWPCI* value of 1 indicates a smooth hole wall profile close to a perfect cylinder geometry.

The Hole Geometry Index *HGI* is an index that combines the values of *HACI* and *HWPCI*, calculated as

$$\mathrm{HGI}=\mathrm{HACI}\times\mathrm{HWPCI} \quad (3)$$

The *HGI* close to 1 indicates a superior hole geometry, meaning that the hole diameter is close to d_e, and the deviation from a straight cylindrical shape is minimal.

The Hole Performance Index *HPI* combines the *HGI* and the ratio of drilling time t_m per unit hole depth h (i.e., the t_m / h).

$$\mathrm{HPI}=\mathrm{HGI}\times(\mathrm{t_m}/\mathrm{h}) \quad (4)$$

If all the measurable parameters are written in an explicit form, then the *HPI* could be given as

$$\mathrm{HPI}=(\mathrm{A_{hole}}/\mathrm{A_e})\times((\mathrm{L_l}+\mathrm{L_r})/2\mathrm{h})\times(\mathrm{t_m}/\mathrm{h}) \quad (5)$$

A low *HPI* indicates good hole geometry (a low *HGI*) and short drilling time (a low t_m / h ratio).

Fig. 3 Parameters of the geometric hole performance evaluation indexes

In this study, the A_e, L_r and L_l were calculated using Autocad 2023 software for each hole drilled in the experiment. Firstly, the holes' cross-sectional images were scanned using a high-resolution scanner, and then each image was transferred to the Autocad program in Jpeg format. The image was scaled using a known length on the real sample, after which the right and left-side wall profiles were manually traced to produce splines. Subsequently, the splines were converted to polylines to determine their lengths. The total length of the polylines was then automatically calculated as wall profile length. For the calculation of A_e, the hole entry and exit diameters were drawn with lines and then joined with the L_r and L_l polylines. Subsequently, the area bounded by the L_r, L_l, hole exit and entry polylines was selected with the "hatch" command, and the A_e value was read from the "properties" tab. All the calculations were transferred to the Excel table with the "list command".

Example calculations for the indices are given here for the hole section shown in Figure 2g. The workpiece material was AISI 1040, and the machining parameters were V_m=4.5 V, n_e=400 rpm, P_f=20 bar, C_e=150 g/L, and h=8 mm. The hole was drilled in 26 seconds (i.e., t_m).

$A_e = d_e \times h = 0.5 \times 8 = 4$ mm^2

$A_{hole} = 7.39$ mm^2

$HACI = A_{hole} / A_e = 7.39 / 4 = 1.848$

$HWPCI = (L_l + L_r) / 2h = (8.29 + 8.33) / (2 \times 8) = 1.039$

$HGI = HACI \times HWPCI = 1.848 \times 1.039 = 1.920$

$HPI = HGI \times (t_m / h) = 1.920 \times (26 / 8) = 6.24$

The study utilized the *HPI* as the primary performance index since it includes all the other performance indexes (given in Eqs. 1-3). The *HPI* represents the hole geometry perfectness as well as the drilling time of the holes for various machining settings and workpiece materials.

3.3. Variation of HPI with process parameters

The study utilized Analysis of variance (ANOVA) to determine the statistically significant machining parameters and their respective percent contribution to the *HPI*. Using a loss function, the Taguchi approach [34, 35] computed the divergence between the desired and experimental values. The signal-to-noise ratio, or S/N ratio, is then created using this loss function.

There are various S/N ratios useable attached to the kind of performance characteristic: lower is better (LB), nominal is best (NB), or higher is better (HB). In ECD, a lower *HPI* indicates better performance. Therefore, LB was selected for the *HPI* to obtain optimum electrochemical drilling performance characteristics. The definitions of the loss function L for electrochemical drilling performance outcomes y_i of n repeated numbers for LB are as follows:

$$L_{LB} = \frac{1}{n}\sum_{i=1}^{n} y_i^2 \tag{6}$$

The Signal-to-noise ratio S/N_{ij} for the i'th performance characteristic in the j'th experiment can be expressed as;

$$S/N_{ij} = -10.\log(L_{ij}) \tag{7}$$

Irrespective of the performance characteristics category, a higher *S/N* value indicates better performance. Thus, the highest *S/N* value represents the optimal process parameter level. The *S/N* values for each experiment, computed using Equations 6 and 7, are presented in Table 4 and Figure 4. As illustrated in Figure 4, the optimal performance for the *HPI* (i.e., the minimum *HPI*) was achieved at level 3 for the material *M* (PM steel), at level 3 for the V_m (6.5 V), at level 2 for the n_e (400 rpm), at level 2 for the P_f (40 bar), and level 3 for the C_e (150 g/L).

Table 4 *S/N* ratios for *HPI*

Level	M	V_m	n_e	P_f	C_e
1	-15.68	-19.98	-17.33	-17.28	-18.06
2	-21.63	-16.11	-16.78	-16.82	-16.90
3	-13.85	-15.06			-16.20
Delta	7.78	4.92	0.55	0.46	1.86
Rank	1	2	4	5	3

Table 4 also shows the difference (Delta) between the process parameters' maximum and minimum *S/N* ratios: 7.78 for *M*, 4.92 for V_m, 1.86 for C_e, 0.55 for n_e, and 0.46 for P_f. The comparison of these values indicates that the highly effective controllable factors on *HPI* were the *M*, with a maximum value of 7.78 (Rank 1) and V_m, with a value of 4.92 (Rank 2). In order of importance, the other factors affecting the *HPI* are C_e, n_e and P_f.

Fig. 4 The effect of process parameters on HPI

The *F*-test allows one to decide with confidence whether the estimates are significantly different [34-35]. A bigger *F* value designates that the change in process parameters remarkably affects performance characteristics. Therefore, the greater the *F*-statistic, the more substantial the evidence of a variation the group means. The relevant confidence table is compared with the F values of the process parameters. When the $F_{\alpha,v1,\ and\ v2}$ value in the confidence table are less

than the F value of the process parameter, this statistically means that the process parameter affects performance characteristics at a very high confidence level. Here, α is risk, v_1 and v_2 are degrees of freedom regarding the numerator and denominator.

Based on the ANOVA results (Table 5), the statistical significance of the difference between group means can be determined by examining the corresponding p-value for the F-statistic. If the p-value is less than 0.05, the ANOVA null hypothesis is rejected, and it is concluded that there is a statistically significant difference between the means of the groups. Suppose the p-value is bigger than or equal to 0.05. In that case, the null hypothesis is not rejected, and it is concluded that there is insufficient evidence to demonstrate a statistically significant difference between the means of the groups. This study's p-value is 0.525 for electrode rotational speed and 0.172 for electrolyte flushing pressure. This indicates insufficient evidence to conclude that the group means for electrode rotational speed and electrolyte concentration differ statistically significantly.

Table 5 Result of ANOVA for *HPI*

Source	DF	Seq SS	Contribution	Adj SS	Adj MS	F-value	p-value
M	2	1322.28	51.92%	1322.28	661.139	116.59#	0.000
V_m	2	594.56	23.34%	594.56	297.278	52.42#	0.000
n_e	1	2.31	0.09%	2.31	2.307	0.41	0.525
P_f	1	10.75	0.42%	10.75	10.748	1.90	0.172
C_e	2	55.67	2.19%	55.67	27.834	4.91#	0.009
Error	99	561.40	22.04%	561.40	5.671		
Total	107	2546.95	100.00%				

at least 99% confidence

Table 5 also shows the percentage distributions of the electrochemical drilling parameters to the *HPI*. The primary factor affecting the *HPI* was the workpiece material, with a 51.92% contribution, followed by the machining voltage, with a 23.34% contribution. The electrolyte concentration, injection pressure, and electrode rotational speed contributions were much lower (2.19%, 0.42%, and 0.09%, respectively).

3.4 Evaluation of experimental findings and HPI

According to Equation 5, the *HPI* is directly proportional to A_{hole}, $L_r + L_l$, and t_m, while A_e and h values remain constant throughout the experiments. Therefore, to minimize the *HPI*, A_{hole}, L_r, L_l, and t_m values should be low. Additionally, the *S/N* ratio analysis in Section 3.3 revealed that the optimum process parameter combination for the minimum *HPI* was the highest settings of

the machining voltage, electrode rotational speed, electrolyte injection pressure and electrolyte concentration, and the use of PM steel workpiece. Thus, it is recommended to use the maximum settings for the machining parameters and the PM steel workpiece to minimize A_{hole}, L_r, L_l, and t_m values. Smaller A_{hole}, L_r, and L_l values represent better hole geometries, while lower t_m values indicate shorter drilling times.

It has been shown that the lowest t_m was achieved when the machining parameters were set at their highest level for the three workpiece materials, as previously mentioned in experimental finding #4 in Section 3.1. Additionally, the *HGI*, which directly impacts the *HPI* value (as shown in Equation 3), plays a significant role in determining if the hole diameter closely matches the electrode diameter d_e and if the deviation from a straight cylindrical shape is minimal. Images of the holes, with a depth of 14 mm, that were drilled in the PM part are displayed in Figures 2i-2k. The hole shown in Figure 2k has the lowest HPI value (3.94), while the hole in Figure 2j ranks second with an HPI of 4.07 (Table 2). Given the similarities in the geometries of the two holes, it can be inferred that the drilling times are also similar. This is supported by the observed drilling times of 28 minutes for the hole in Figure 2j and 24 minutes for the hole in Figure 2k. The hole in Figure 2i has an *HGI* value (2.37) that resembles those in Figures 2j and 2k. However, its higher HPI value (9.83) indicates a longer drilling time (58 minutes) than the other two. Figures 2a-2d depict the holes with a depth of 8 mm that were drilled in Hadfield steel. These holes' close *HGI* values (2.78, 3.38, 3.73, and 3.62, respectively) confirm their geometric similarities. These holes' high *HPI* values (20.59, 22.82, 21.04, and 20.41, respectively) suggest that they required long drilling times (59 min, 54 min, 45 min, and 45 min, respectively). On the other hand, the hole drilled in the same material (Figure 2h) with a much lower *HGI* (1.72) and *HPI* (8.61) implies a superior hole geometry and shorter drilling time (32 minutes) compared to the others.

So, the experimental findings discussed in Section 3.1 are represented in the *HPI* in terms of A_{hole}, L_r, L_l, and t_m. For example,

-"localized diameter variations and non-uniform wavy wall geometries" are represented in terms of A_{hole}, L_r and L_l,

-"hole diameters larger than the electrode diameter" and "hole diameters increased for Hadfield and AISI 1040 steels but decreased for PM steel with increasing electrode rotational speed" are represented in terms of A_{hole},

-"the drilling time decreased as the machining voltage, electrolyte concentration, electrode flushing pressure, and electrode rotational speed increased", "the hole drilling time of the PM steel was less than that of the AISI 1040 and Hadfield steel samples", and "the Hadfield steel had the longest drilling times" are represented in terms of t_m in the *HPI*.

These findings demonstrate that the *HPI* is a suitable tool for modeling experimental results related to hole geometry and drilling time.

3.5 Confirmation experiments

The confirmation experiment tests a particular combination of the factors and levels previously assessed to validate the conclusions drawn during the analysis phase. This paper reports on a novel experiment planned and carried out to identify the ideal levels of machining parameters. The last phase involves predicting and verifying the enhancement of performance characteristics. The predicted *S/N* ratio $\hat{\eta}$ can be calculated using the ideal levels of electrochemical drilling parameters.

$$\hat{\eta} = \eta_m + (\sum_{i=1}^{k} (\bar{\eta}_i - \eta_m) \quad (8)$$

Here, the S/N_m is the total mean of the *S/N* ratio, $\bar{\eta}_i$ the mean of the *S/N* ratio at the ideal level, and *k* is the number of the main process factors significantly affecting performance.

Table 6 presents the results of experimental verification using optimal electrochemical drilling factors. It compares the predicted *HPI* with the actual *HPI* using the optimal process factors. The *S/N* ratio for *HPI* improved by 8.12 dB from the starting electrochemical drilling factors to the optimal ones, while the *HPI* decreased by about 2.54 times. The approach used significantly improved the *HPI*. The experimental results have confirmed the currency of the Taguchi approach for improving electrochemical drilling performance and optimizing electrochemical drilling factors.

Table 6 The confirmation experiment for *HPI*

	Starting machining parameters	Optimal machining parameters	
		Prediction	Experiment
Level	$M2V_m2n_e2P_f2C_e2$	$M3V_m3n_e2P_f2C_e3$	$M3V_m3n_e2P_f2C_e3$
HPI	9.9360	1.9037	3.9003
S/N ratio for *HPI* (dB)	-19.9442	-5.5919	-11.8219
Improvement *S/N* ratio for *HPI* = 8.12 dB			

4. Conclusion

This study introduced a novel performance index, *HPI*, in terms of electrode diameter, hole depth, hole cross-sectional area, the right- and left-side wall profile length, and hole drilling time to assess the perfection of hole geometry and drilling time in the electrochemical drilling (ECD) process. The *HPI* proved a valuable metric for evaluating these factors under different machining parameters and workpiece materials. Lower *HPI* values indicated superior hole wall profiles, closer alignment with electrode area, and shorter drilling times. Through signal-to-noise ratio analysis, it was determined that the optimal combination of process parameters for achieving the lowest *HPI* involved high settings for machining voltage, electrode rotational speed, electrolyte injection pressure, electrolyte concentration, and using PM steel as the workpiece material. Additionally, it was observed that workpiece material and machining voltage significantly influenced *HPI*, with contributions of 51.92% and 23.34%, respectively. The electrode rotational speed, electrolyte injection pressure, and electrolyte concentration were less effective for *HPI*, with a 2.19%, 0.42%, and 0.09% contribution, respectively. The proposed statistical technique successfully reduced *HPI* by 2.2 times from initial machining parameters to optimal settings, demonstrating the effectiveness of the Taguchi approach in enhancing machining efficiency and optimizing ECD operations.

Future studies could explore lower settings of electrode rotational speed, electrolyte injection pressure, and electrolyte concentration to achieve satisfactory hole geometry and drilling speed with reduced energy and salt consumption, as these parameters were found to have minimal impact on *HPI* in this study.

References

[1] Ahn SH, Ryu SH, Choi D K, Chu CN (2004) Electro-chemical micro drilling using ultra short pulses. Precision Engineering 28(2):129-134.
[2] Fan ZW, Hourng LW (2009) The analysis and investigation on the microelectrode fabrication by electrochemical machining. Int J Mach Tools and Manuf 49(7-8):659-666.
[3] Zhu D, Wang W, Fang XL, Qu NS, Xu ZY (2010) Electrochemical drilling of multiple holes with electrolyte-extraction. CIRP annals 59(1):239-242.
[4] Wang W, Zhu D, Qu N, Huang S, Fang X (2010) Electrochemical drilling inclined holes using wedged electrodes. Int J Adv Manuf Technol 47:1129–1136.
[5] Wang W, Zhu D, Qu NS, Huang SF, Fang XL (2010) Electrochemical drilling with vacuum extraction of electrolyte. J Mat Proc Tech 210(2): 238-244.
[6] Fan ZW, Hourng LW (2011) Electrochemical micro-drilling of deep holes by rotational cathode tools. Int J Adv Manuf Technol 52:555–563.
[7] Fan ZW, Hourng LW, Lin MY (2012) Experimental investigation on the influence of electrochemical micro-drilling by short-pulsed voltage. Int J Adv Manuf Technol 61:957–966.
[8] Mithu MAH, Fantoni G, Ciampi J (2011) The effect of high frequency and duty cycle in electrochemical microdrilling. Int J Adv Manuf Technol 55:921–933.
[9] Choi SH, Kim BH, Shin HS, Chu, CN (2013) Analysis of the electrochemical behaviors of WC–Co alloy for micro ECM. J Mat Proc Tech 213(4):621-630.
[10] Fang X, Qu N, Li H, Zhu D (2013) Enhancement of insulation coating durability in electrochemical drilling. Int J Adv Manuf Technol 68:2005–2013.
[11] Wang J, Chen W, Gao F, Han F (2014) A new electrode sidewall insulation method in electrochemical drilling. Int J Adv Manuf Technol 75:21–32.
[12] Zeng Y, Fang X, Zhang Y, Qu N (2014) Electrochemical drilling of deep small holes in titanium alloys with pulsating electrolyte flow. Advances in Mechanical Engineering 6:167070.
[13] Chandrasekhar S, Prasad, NBV (2019) Experimental Parametric Investigation of Electrochemical Drilling of AA6061-TiB2 In Situ Composites. J Inst of Eng (India) Series C 100(5):771-778.
[14] Hung JC, Liu YR, Tsui, HP, Fan ZW (2019) Electrode insulation layer for electrochemical machining fabricated through hot-dip aluminizing and microarc oxidation on a stainless-steel substrate. Surf and Coat Tech 378: 124995.
[15] Sharma S, Jain V, Shekhar R (2002) Electrochemical Drilling of Inconel Superalloy with Acidified Sodium Chloride Electrolyte. Int J Adv Manuf Technol 19:492–500.
[16] Bilgi DS, Jain VK, Shekhar R, Mehrotra S (2004) Electrochemical deep hole drilling in super alloy for turbine application. J of Mat Proc Tech 149(1-3): 445-452.
[17] Bilgi DS, Jain VK, Shekhar R, Kulkarni AV (2007) Hole quality and interelectrode gap dynamics during pulse current electrochemical deep hole drilling. Int J Adv Manuf Technol 34:79–95.
[18] Jain NK, Potpelwar A, Pathak S, Mehta NK (2016) Investigations on geometry and productivity of micro-holes in Incoloy 800 by pulsed electrolytic jet drilling. Int J Adv Manuf Technol 85: 2083–2095
[19] Zhang Y, Qu N, Fang X, Wang X (2019) Eliminating spikes by optimizing machining parameters in electrochemical drilling. J Manuf Proc 37:488-495
[20] Ali S, Hinduja S, Atkinson J, Pandya M (2009) Shaped tube electrochemical drilling of good quality holes. CIRP Annals 58(1):185-188
[21] Zare Chavoshi S (2011) Analysis and predictive modeling of performance parameters in electrochemical drilling process. Int J Adv Manuf Technol 53:1081–1101
[22] Tajdari M, Chavoshi SZ (2013) Prediction and Analysis of radial overcut in holes drilled by electrochemical machining process. C Euro J Eng 3:466–474.
[23] Baoji M, Cheng P, Yun K (2018) Effect of power signal waveform on shape accuracy in electrochemical drilling. In IOP Conference Series: Mat Sci and Eng 392(3):032015 IOP Publishing
[24] Natarajan M, Kumanan S, Sathiyanarayanan C (2015) Prediction of Material Removal Rate in Electro Chemical Machining using Multiple Regression Analysis. Int Conf Adv Prod Ind Eng 250-256

[25] Deka H, Fangueiro R (2016) Influence of electrolyte composition on the performance of electrochemical micromachined deep holes. Int J Adv Man Tech 82(5-8):1643-1653
[26] Yang X, Li W, Li K, Wang M, Xiu C (2021) Experimental investigation on electrochemical discharge machining drilling process of small holes on zirconium dioxide ceramic. Cer Inter, 47(1): 711-719
[27] Safaei S, Beheshtian J, Saraeian P (2021) Investigating the effect of sodium hydroxide concentration on the electrochemical drilling process of Ti6Al4V alloy. J Manuf Proc 63:1133-1141
[28] Kumar A, Kumar N, Kumar A (2023) Experimental study on electrochemical drilling of Ti6Al4V titanium alloy using Taguchi method. Mat Tod: Proc 42:2286-2293
[29] Özerkan HB, Çoğun C (2020) Electrochemical Small Diameter Deep Hole Drilling of Powder Metal Steel. Trans FAMENA 44(4):47-58. doi.org/10.21278/TOF.444007919
[30] Ayhan E, Yurdakul M, Çoğun C, iç YT (2023) The entropy method integrated RSM model to evaluate hole geometries in electrochemical blind hole drilling. Aust J Mech Eng, 1-19. https://doi.org/10.1080/14484846.2023.2287259.
[31] Çoğun C, Deniz, TÇ, Küçüktürk G (2012) A geometrical approach for reduction of tool shape degeneration in electric discharge machining (EDM). Mach Sci Tech 16(3):445-458
[32] Özerkan HB, Çoğun C (2022) Design and implementation of an electrode feed rate control system in the electrochemical drilling process. J Braz S Mech Sci Eng 44(9):385
[33] Sharma S, Jain VK, Shekhar R (2022) Electrochemical Drilling of Inconel Superalloy with Acidified Sodium Chloride Electrolyte. Int J Adv Man Tech 19:492–500
[34] Taguchi G, Chowdhury S, Wu Y (2004) In Taguchi's Quality Engineering Handbook. New Jersey, USA.
[35] Tosun G, Tosun N (2012) Analysis of Process Parameters for Porosity in Porous NiTi Implants. Mat Manuf Proc 27(11):1184–1188. https://doi.org/10.1080/10426914.2011.648692

Funding: This research was supported by the Basic Science Research Program through the National Research Foundation of Korea (NRF) funded by the Ministry of Education (2020R1I1A3052258).

Data availability

Data sharing does not apply to this article as no datasets were generated or analyzed during the current study.

Declarations Conflict of interest

There is no conflict of interest.

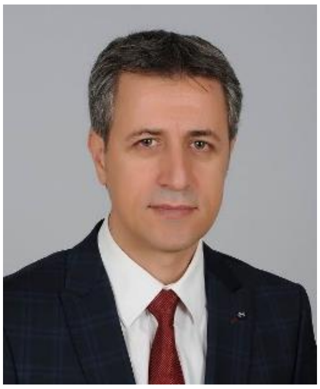

Nihat TOSUN received a doctorate degree from Firat University in 2001. He is working as a professor at the Mechanical Engineering Department in the Engineering Faculty of Firat University in Turkey. His research interests include Manufacturing Technologies, Material Engineering, CNC and CAD/CAM etc.

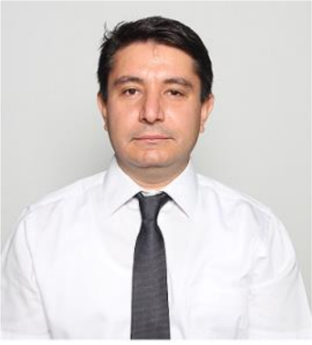

H. Bekir Özerkan received his B.Sc. and Ph.D. degrees from the Gazi University of Faculty of Engineering in 2003 and 2010, respectively. He is currently an associate professor in Faculty of Engineering, Ankara, Turkey. His research interests include Conventional and Nontraditional Manufacturing Processes, Machine Design.

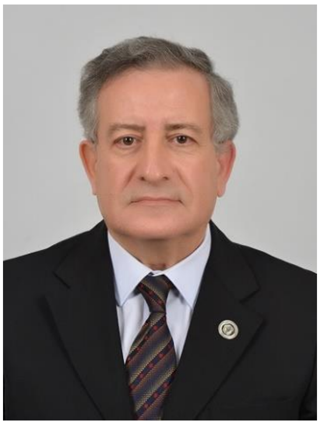

Can ÇOĞUN received the doctorate degree from Middle East Technical University in 1987. He is working as a professor at Mechatronics Engineering Department in Engineering Faculty of Çankaya University in Turkey. His research interests include Nontraditional Manufacturing Processes (especially Electric Discharge Machining), Chip removal Processes, Micromachining Processes.

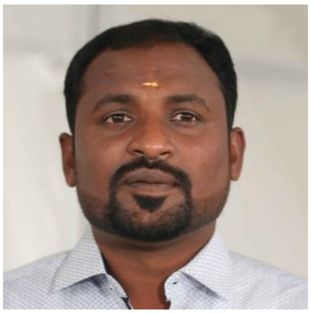

Raja Venkatesan received his Ph.D. in Polymer Chemistry (2018) from Anna University in Chennai, India. He currently has a position with Yeungnam University in South Korea as a Research Professor. His areas of specialization are food packaging and thermoelectric applications with composite blends and polymer synthesis.

Figure

Click here to access/download;Figure;Fig1.tif

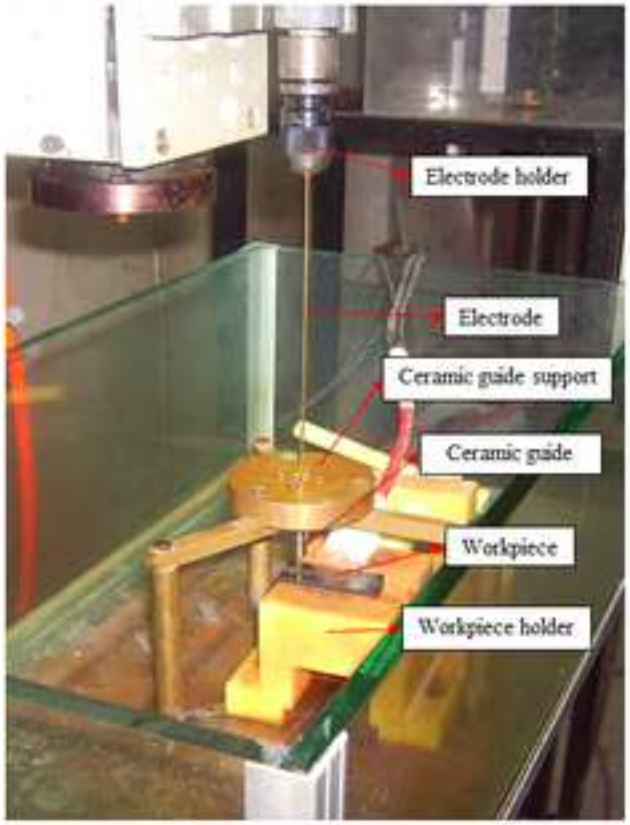

Figure

Click here to access/download;Figure;Fig2.tif

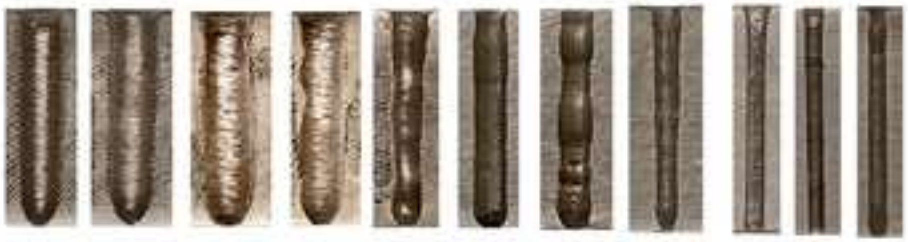

(a) (b) (c) (d) (e) (f) (g) (h) (i) (j) (k)

 Click here to access/download;Figure;Fig3.tif

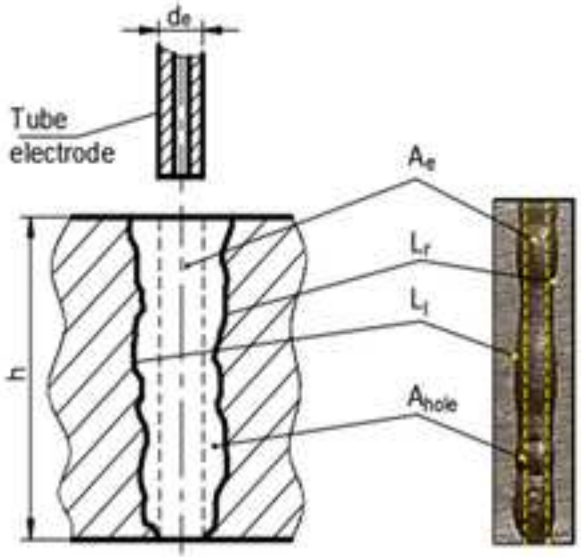

Click here to access/download;Figure;Fig4.tif

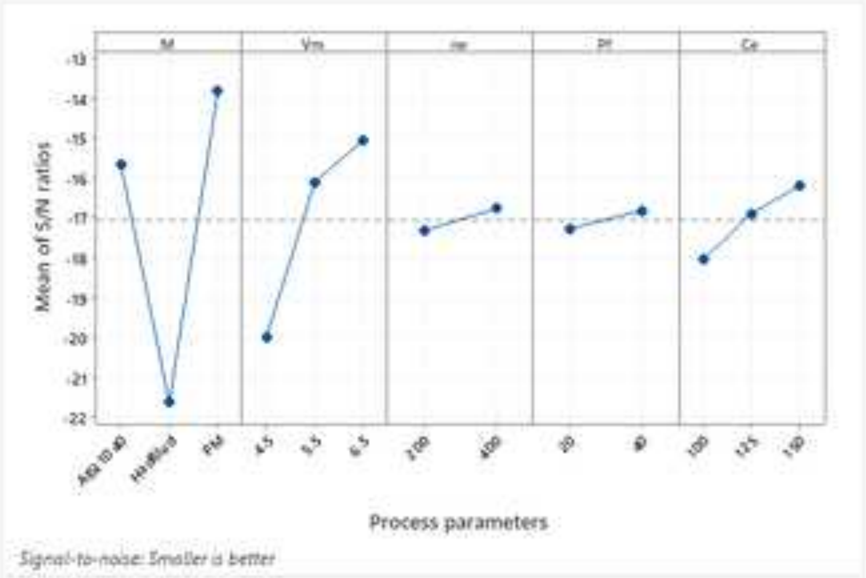

Supplement: Attachment — Submitted filename: PONE-D-24-24552.pdf [file pone.0314236.s001.pdf]
