# Supplementary material for: A novel hole performance index to evaluate the hole geometry and drilling time in the electrochemical drilling process
Source: PLoS One. 2024 Dec 3;19(12):e0314236. doi: 10.1371/journal.pone.0314236 (PMC11614253; doi:10.1371/journal.pone.0314236)
Supplement: Attachment — Submitted filename: PLOS-Responses to Reviewers comments (2 3 and 4).pdf [file pone.0314236.s003.pdf]

Reviewers' Comments	Response to Reviewers
**Reviewer #1:**	*Reviewer #1's comments are responded to in the attached file entitled "Responses to the Reviewer 1 Comments".*
**Reviewer #2:**<br>The authors have done a good work and its focus on dimension analyses of the machined hole. There are few queries authors need to clarify	
Replace the figures with sharp resolution, figure 1 is blurred.	*Thank you for your valuable feedback. Image quality is slightly degraded when the manuscript is converted to PDF. The highest quality images available have been used in the manuscript.*
Do you have SEM image of drilled hole, add the same.	*Thank you for your valuable feedback. The study did not include surface topography, microstructure, or morphology imaging of the material. Therefore, SEM analysis was not performed.*
what is the thickness of the workpiece is all material of same size?	*No, the thicknesses of the samples are different. The dimensions of the samples are presented in the third paragraph of the "Materials" section.*
what is purpose of measuring the time and i don't see any output performance value related to time?	*Thank you very much. The main objective of the ECD method is to obtain the desired hole geometries in the shortest possible drilling time. The main objective of this study is to evaluate the hole geometry using the newly generated "Hole Performance Index, HPI," which incorporates the drilling time. Thus, the "hole drilling time" is measured and used in the HPI equation (the fourth and fifth equations of the "Definition of Hole Geometry and Drilling Performance Evaluation Indexes" sub-heading of the "Experimental Procedure" section).*
How did you ensure the completion of hole drilling process completion?	*Thank you for your valuable assessment. In the tests, the drilling process is completed when the electrolyte is blown out of the bottom of the workpiece, and the machining voltage drops to zero.*
How did you measure the hole circumference waviness?	*Thank you for your valuable assessment. The measurement method was explained under the title "Measurement Techniques."*
Provide the details of instruments for evaluation of output performance?	*Thank you for your valuable comment. The required information is added to the manuscript (the first paragraph of the "Methods" section and the second paragraph of the "Measurement Techniques" section.)*
Based on ANOVA you suggest material and voltage has significant influence. Why? Based on the ECM principle the material dissolution takes place at faster rate irrespective of the material. Justify with literature	*Thank you for your valuable contribution. Two sentences were added to the manuscript (the last two sentences of the subsection "Variation of HPI with process parameters" under the main section "Results") to clarify the situation.*
Add the reference with<br>doi.org/10.3103/S1068375517050143<br>doi.org/10.1080/10426914.2022.2030874,<br>10.4314/bcse.v37i5.17	*The references were used in the literature review section (Introduction).*

**Reviewer #3:**<br>1. What material is the electrode made of (chemical composition)?	*Thank you. The information requested was given in the 2nd paragraph of the "Materials" subsection.*
2. What shape does the electrode have (dimensions)?	*Thank you. The information requested was given in the 2nd paragraph of the "Materials" subsection.*
3. How did the authors analyze the thermal changes and cracks that occurred?	*Thank you for your valuable assessment. Thermal changes and cracks rarely occur in ECD under high voltage and feed rate settings as well as poor electrolyte flushing conditions. So, thermal changes and cracks were not examined under the used machining settings.*
4. Please show a diagram of electrochemical hole machining (ESD).	*A schematic diagram of the ECD process has been added to the Introduction section.*
5. What electrolytes were used in the studies?	*Thank you for your constrıctive criticism. The electrolyte type and concentrations were mentioned in the second paragraph of the "Materials" section.*
6. How to measure Lr and Ll? Please insert a drawing. How was the hole axis determined?	*The L_r and L_l measurements are explained in the first paragraph of the "Measurement Techniques" section using Figure 3. Determining the "hole axis" was unnecessary to analyze the hole geometry.*
7. Please describe the methodology for measuring the hole.	*Thank you for your valuable assessment. The measuring hole methodology was described in the first paragraph of the "Measurement Techniques."*
8. How did the authors take into account the influence of electrode wear?	*Thank you for your input. The last sentence of result #2 in the "Experimental hole geometries" section mentioned "no tool wear".*
9. How many repetitions of the study were performed by the authors? What research plan did the authors adopt?	*Thank you for your valuable assessment. The drilling experiment was performed three times to ensure the reliability of the results and the measurements were averaged. A full factorial design of experiments was used to evaluate the effect of workpiece materials and machining parameters on HPI (Table 2). This information is given in the last two sentences of the first paragraph of the "Experimental Procedure" subsection of the "Methods" section.*
10. Please compare the proposed indicator with indicators described in the literature. It is necessary.	*A paragraph before the last paragraph of the heading "Definition of Hole Geometry and Drilling Performance Evaluation Indexes" (in the Materials and Methods section), comparing the indicators used in the literature and the HPI, is added to the manuscript*
11. How will the adopted HPI distinguish the slenderness of the hole from the ovality?	*Thank you very much for your contribution. There is no direct term in the HPI to distinguish the slenderness of the hole from the ovality. However, the A_{hole}/A_e ratio in the HPI formulation (eq. 5) indicates the slenderness of the hole. The smaller the ratio, the more slender the hole. The ovality (conicity) of the hole could have been determined by the ratio of the hole entrance diameter to the exit*

	diameter and incorporating this into the HPI formulation.
**Reviewer #4:** The author has done the research entitled "A NOVEL HOLE PERFORMANCE INDEX TO EVALUATE THE HOLE GEOMETRY AND DRILLING TIME IN THE ELECTROCHEMICAL DRILLING PROCESS". This paper examines the impact of process variables, such as machining voltage, electrolyte concentration, electrode rotational speed, electrolyte flushing pressure, and workpiece material, on the novel hole performance index HPI in the electrochemical drilling process. The manuscript has strong technical content. The tests were also carried out scientifically. With major changes, this manuscript will be accepted. The following major corrections must be made.<br>1. The expansion of HGI is not listed in the nomenclature section which may confuse the reader. It should be added.	*Thank you very much. The HGI and its expansion are added to the nomenclature.*
2. In Table 2, some of the process parameters are taken into account as two levels, some are three levels. Justify in depth the selection of process parameters and their corresponding levels, providing a comprehensive explanation.	*Thank you very much. The justification of selection of the machining parameters is given in the first paragraph of the "Methods" section in detail.*
3. In Table 3, the remarks column does not explore valuable data. It should be thoroughly revised and rewritten with some statements. If it is not the most important, you can delete that particular column.	*Thank you very much. You are quite right. We have deleted the column entirely, as the column information is included in the caption of Figure 3.*
4. In the result and discussion, the author has discussed only HPI, not HGI. Justify.	*Thank you very much for your valuable comment. In the last two sentences of the heading "Definition of Hole Geometry and Drilling Performance Evaluation Indexes" (under the "Materials and Methods" section), we mentioned that only the HPI was used as the performance index throughout the study since it covers the HGI.*
5. All figures are not clear and the quality of the figure is very poor.	*Thank you for your valuable assessment. Image quality decreases slightly when the manuscript is converted to PDF. The highest quality images that can be obtained were used in the manuscript.*
6. How to find out the percentage of contributions for each process parameter in Table	*The percent contribution is obtained by summing all the sum of squares (SS) and then dividing each individual SS by dividing by the total SS and multiplying by 100. This sentence has been added to the relevant section below Table 4.*
5. Also, the process parameters like ne, Pf, and Ce have not contributed much. Justify.	*Thank you for your valuable assessment. The most effective parameters were calculated statistically according to the experimental results and ANOVA analysis performed with the selected parameters. The effect of the input variables you specified was less than the others.*
7. The author should include the microstructural images (OM or SEM) at least the optimized conditions	*Thank you. The study did not include surface topography, microstructure, or morphology*

	imaging of the drilled holes. Therefore, no OM or SEM images are available.
