# Supplementary material for: A novel hole performance index to evaluate the hole geometry and drilling time in the electrochemical drilling process
Source: PLoS One. 2024 Dec 3;19(12):e0314236. doi: 10.1371/journal.pone.0314236 (PMC11614253; doi:10.1371/journal.pone.0314236)
Supplement: Attachment — Submitted filename: Review comments.docx [file pone.0314236.s002.docx]

The author has done the research entitled “**A NOVEL HOLE PERFORMANCE INDEX TO EVALUATE THE HOLE GEOMETRY AND DRILLING TIME IN THE ELECTROCHEMICAL DRILLING PROCESS**”. This paper examines the impact of process variables, such as machining voltage, electrolyte concentration, electrode rotational speed, electrolyte flushing pressure, and workpiece material, on the novel hole performance index HPI in the electrochemical drilling process. The manuscript has strong technical content. The tests were also carried out scientifically. With major changes, this manuscript will be accepted. The following major corrections must be made.

1. The expansion of HGI is not listed in the nomenclature section which may confuse the reader. It should be added.
2. In Table 2, some of the process parameters are taken into account as two levels, some are three levels. Justify in depth the selection of process parameters and their corresponding levels, providing a comprehensive explanation.
3. In Table 3, the remarks column does not explore valuable data. It should be thoroughly revised and rewritten with some statements. If it is not the most important, you can delete that particular column.
4. In the result and discussion, the author has discussed only HPI, not HGI. Justify.
5. All figures are not clear and the quality of the figure is very poor.
6. How to find out the percentage of contributions for each process parameter in Table 5. Also, the process parameters like n_e_, P_f,_ and C_e_ have not contributed much. Justify.
7. The author should include the microstructural images (OM or SEM) at least the optimized conditions.
