# Supplementary material for: A novel hole performance index to evaluate the hole geometry and drilling time in the electrochemical drilling process
Source: PLoS One. 2024 Dec 3;19(12):e0314236. doi: 10.1371/journal.pone.0314236 (PMC11614253; doi:10.1371/journal.pone.0314236)
Supplement: Attachment — Submitted filename: Responses to the Reviewer-1 Comments.docx [file pone.0314236.s004.docx]

|  | **Reviewer #1: Comments** | **Author responses** |
| --- | --- | --- |
| **1-** | (*For abstract*)  very long sentences...authors are advised to see the entire mansucript and revise it properly...such long sentences must not be in the paper | Thank you. The long sentences are revised. |
| **2-** | (*For nomenclature*)  see nomenclature section properly.. | Thank you. We reworked the "nomenclature" section. |
| **3-** | (*For introduction part*)  No reference...also objectives must be written properly and it must not be in first paragraph.. | The simple/basic information given in the first paragraph is a well-known fact about the ECD process for the researchers and readers in the field. So, with all our respect, the authors prefer not to give reference(s) in this part.  A separate section, named "objectives," is added to the manuscript following the "introduction" section. |
| **4-** | (*For literature survey findings*)  it is not written properly... | Thank you. This part is rewritten. |
| **5-** | (*For experiments part*)  is it proper...where is materials and method section... | Thank you for your constructive criticism. The "materials and methods" section is added. |
| **6-** | (*For Fig 3.)*  needs to be revised...explanation is not proper | The caption is rewritten. |
| **7-** | (*For “Confirmation experiments part”)*  wrong one...in place of confirmation, valdiation is a proper word and need to be explained properly about the variations too..authors are instructed to revise properly... | The "confirmation" wording is an almost standart usage for the ANOVA and related statistical analyses. Anyway, we have changed the word "confirmation" to "validation". |
| **8-** | (*For “Conclusion” part*)  Conclusion is poor...authors are instructed to add the conclusion properly..it looks like pargraph | The conclusion section is reworked. |
