# Supplementary material for: A novel hole performance index to evaluate the hole geometry and drilling time in the electrochemical drilling process
Source: PLoS One. 2024 Dec 3;19(12):e0314236. doi: 10.1371/journal.pone.0314236 (PMC11614253; doi:10.1371/journal.pone.0314236)
Supplement: Attachment — Submitted filename: Responses to the Reviewer-1 Comments.docx [file pone.0314236.s005.docx]

**REVISION 2 COMMENTS**

|  | **Reviewer #1: Comments** | **Author responses** |
| --- | --- | --- |
| **1-** | (*For abstract and conclusion*)  see the comments given during first revision and revise properly. Still abstract and conclusion have not rewritten properly. | Thank you. The comments in the first revision have been revised properly. The abstract and conclusion sections have been revised and rewritten. |

**REVISION 1 COMMENTS**

|  | **Reviewer #1: Comments** | **Author responses** |
| --- | --- | --- |
| **1-** | **(*For abstract*)**  **very long sentences...authors are advised to see the entire mansucript and revise it properly...such long sentences must not be in the paper** | **Thank you. The long sentences are revised.** |
| **2-** | **(*For nomenclature*)**  **see nomenclature section properly..** | **Thank you. We reworked the "nomenclature" section.** |
| **3-** | **(*For introduction part*)**  **No reference...also objectives must be written properly and it must not be in first paragraph..** | **The simple/basic information given in the first paragraph is a well-known fact about the ECD process for the researchers and readers in the field. So, with all our respect, the authors prefer not to give reference(s) in this part.**  **A separate section, named "objectives," is added to the manuscript following the "introduction" section.** |
| **4-** | **(*For literature survey findings*)**  **it is not written properly...** | **Thank you. This part is rewritten.** |
| **5-** | **(*For experiments part*)**  **is it proper...where is materials and method section...** | **Thank you for your constructive criticism. The "materials and methods" section is added.** |
| **6-** | **(*For Fig 3.)***  **needs to be revised...explanation is not proper** | **The caption is rewritten.** |
| **7-** | **(*For “Confirmation experiments part”)***  **wrong one...in place of confirmation, valdiation is a proper word and need to be explained properly about the variations too..authors are instructed to revise properly...** | **The "confirmation" wording is an almost standart usage for the ANOVA and related statistical analyses. Anyway, we have changed the word "confirmation" to "validation".** |
| **8-** | **(*For “Conclusion” part*)**  **Conclusion is poor...authors are instructed to add the conclusion properly..it looks like pargraph** | **The conclusion section is reworked.** |
